# Formation of multimeric antibodies for self-delivery of active monomers

**DOI:** 10.1080/10717544.2016.1242179

**Published:** 2017-02-03

**Authors:** Yaron Dekel, Yossy Machluf, Tal Gefen, Gennady Eidelshtein, Alexander Kotlyar, Yaron Bram, Ehud Shahar, Farah Reslane, Elina Aizenshtein, Jacob Pitcovski

**Affiliations:** 1 Shamir Research Institute, University of Haifa, Kazrin, Israel,; 2 Department of Clinical Laboratory, Zefat Academic College, Zefat, Israel,; 3 Department of Life Sciences, Tel Hai College, Upper Galilee, Israel,; 4 Consultant, specialist in the fields of biochemistry, molecular biology and genetics,; 5 MIGAL – Galilee Technology Center, Kiryat Shmona, Israel,; 6 Department of Biochemistry and Molecular Biology, Tel Aviv University, Tel Aviv, Israel, and; 7 Department of Molecular Microbiology and Biotechnology, Tel Aviv University, Tel Aviv, Israel

**Keywords:** Protein drug release, bioactivity, antibodies, multimers, self-delivery

## Abstract

Proteins and peptides have been used as drugs for almost a century. Technological advances in the past 30 years have enabled the production of pure, stable proteins in vast amounts. In contrast, administration of proteins based on their native active conformation (and thus necessitating the use of subcutaneous injections) has remained solely unchanged. The therapeutic anti-HER2 humanized monoclonal immunoglobulin (IgG) Trastuzumab (Herceptin) is a first line of the treatment for breast cancer. Chicken IgY is a commercially important polyclonal antibody (Ab). These Abs were examined for their ability to self-assemble and form ordered aggregates, by several biophysical methods. Atomic force microscopy analyses revealed the formation of multimeric nanostructures. The biological activity of multimeric IgG or IgY particles was retained and restored, in a dilution/time-dependent manner. IgG activity was confirmed by a binding assay using HER2 + human breast cancer cell line, SKBR3, while IgY activity was confirmed by ELISA assay using the VP2 antigen. Competition assay with native Herceptin antibodies demonstrated that the binding availability of the multimer formulation remained unaffected. Under long incubation periods, IgG multimers retained five times more activity than native IgG. In conclusion, the multimeric antibody formulations can serve as a storage depositories and sustained-release particles. These two important characteristics make this formulation promising for future novel administration protocols and altogether bring to light a different conceptual approach for the future use of therapeutic proteins as self-delivery entities rather than conjugated/encapsulated to other bio-compounds.

## Introduction

Advances in protein production technologies have enabled the production of vast amounts of pure, stable and active proteins. Proteins and peptides, such as insulin, growth hormones, antibodies and enzymes, are being routinely used for therapeutic purposes (Szlachcic et al., 2011; Ansari et al., 2016). However, in contrast to these advances in protein production and purification techniques, the mode of administration of proteins to patients has remained principally unchanged, based almost solely on injections of the proteins in their native conformation (Narayanaswamy et al., 2016).

A crucial aspect of native protein formulations is their *in vitro* stability (Gimeno et al., 2002). Proteins in their native conformation tend, with time, to misfold and to aggregate, reaching a more favorable thermodynamic state. This state bears lower energy levels and thus is unavoidable. In the form of amorphous aggregates, proteins lose their native conformation, sediment and consequently lose their biological activity. The process is usually detrimental (Gazit, 2002). Various commercial formulations have been developed to overcome this problem, some of them containing toxic irritants with possible carcinogenic effects (Rajpar et al., 2006; Pfohler et al., 2008; Danne & Bolinder, 2011; Teng et al., 2011). A protein formulation, free of chemical additives, that can serve as a depot of active therapeutic proteins, protect the aggregated proteins and provide a sustained release mechanism, may reduce the amount of injections or even transform veteran administration protocols into “patient friendly” ones, and therefore is of high interest.

Antibodies and antibody-derived molecules are the fastest growing class of biopharmaceutical products (Chames et al., 2009). Accumulating evidence reveals the self-assembly mechanisms of high-molecular-weight proteins (HMWPs), such as antibodies. Studies on the nature of IgG aggregation have suggested that soluble IgG aggregates are composed of monomers, in which at least some fraction of the protein chain has adopted an amyloid-like structure, similar to that of single-domain proteins (Brummitt et al., 2011a,b). The ability of HMWPs (in the range of 50 kD) to form fibrils *in vitro* was demonstrated recently. These fibrils, albeit poor, were stained with common fibrillation dyes, such as Congo red or Thioflavin-T (ThT), hence leading to the assumption that these fibrils are mostly non-amyloidogenic (Ramshini et al., 2011).

The aggregation stability of IgG monoclonal antibodies was also studied. At pH lower than 4.0, addition of salt induces a reversible aggregation to oligomers accompanied by an increase in the content of the β-sheet structure (Arosio et al., 2011). Significant differences in both oligomerization and growth rates were found for different antibodies, even those belonging to the same subclass, as was shown for IgG1 and IgG2 (Arosio et al., 2013; Nicoud et al., 2014a). Cosolutes, such as NaCl or sorbitol, were shown to accelerate and inhibit, respectively, the aggregation kinetics of monoclonal antibodies. In the case of IgG1, NaCl accelerated aggregation events, while sorbitol inhibited protein unfolding, making the monomer unfolding phase the rate limiting step of the reaction. In the case of IgG2, NaCl and sorbitol affected all four aggregation phases to the same extent (Nicoud et al., 2014b). A novel model of the aggregation kinetics of monoclonal antibodies under thermal stress (70 °C) and a wide range of protein concentrations was recently suggested (Nicoud et al., 2016). Tetramers are assumed to be the largest reversible species (Nicoud et al., 2014a).

In contrast to HMWPs (Whittingham et al., 2002), small peptides, such as the major therapeutic peptide insulin, can restore their biological activity following fibrillar dissociation. Protein fibrils were used in an attempt to deliver insulin orally (Dekel et al., 2010). Insulin was driven to its fibrillar state inside different micro-particles. These fibril formulations were proved to be resistant to various stimulatory conditions that are found in the gastrointestinal tract. Moreover, these fibril formulations were therapeutically active *in vivo* (Dekel et al., 2010). Thus, protein fibrillation is not always a detrimental process. In some hormone-secreting cells in the rat brain, hormones are stored in their fibrillar form, and are released as active monomers from the fibrils, demonstrating the importance of functional fibrils. Thus, in the hippocampus protein fibrils can be an indicator of disease, like in the case of Alzheimer, while in the pituitary they may be part of natural processes and functions (Maji et al., 2009). Another example of functional fibrils is the case of peptide hormones in secretory granules that possess highly regulated storage function (Kumar et al., 2016).

This study is aimed at examining the ability of HMWPs to self-assemble while retaining their active biological properties. We examined the ability of the 150 kD IgG Trastuzumab (Herceptin), as a model for the class of (humanized) therapeutic antibodies, to self-aggregate under controlled conditions and regain its bioactive properties upon dissociation. Herceptin is a recombinant humanized monoclonal antibody against HER2/Neu receptor, which is the first line treatment for Her2 + breast cancer patients and thus of general interest (De Mattos-Arruda & Cortes, 2012). Alongside this IgG, another immensely studied and useful HMWP, the 170 kD chicken IgY was examined. Human Insulin, which was vastly studied under aggregated state, was used here as a benchmark protein, which presents the familiar behavior of small peptides under aggregative conditions. This report will cover the process of IgG and IgY self-assembly into multimers and examine the conditions under which these particles dissociate back into active antibodies *in vitro*, demonstrating a novel concept of self-delivery proteins.

## Methods

### IgG or IgY self-assembly preparation, recording and imaging

Humanized monoclonal anti-HER2 IgG (Roche, Basel, Switzerland) or chicken IgY (following production and purification protocols which were described in details elsewhere; Yosipovich et al., 2015), at 1 mg/ml, were acidified to pH 2 at 37 °C and agitated or stirred (hereafter referred to as “self-assembled Ig”). Self-assembled and native IgGs or IgYs were monitored by:

- *ThT fluorescence*: It was measured with a quartz cuvette in a Varian-Cary eclipse fluorometer (Sigma Chemical Co., St. Louis, MO; Ex. 450 nm, Em. 460–600 nm, peak at 482 nm) at 100 μM ThT. ThT working concentration was determined after various trials at different concentrations, from 0.5 to 500 μM (data not shown). Five hundred microliters of ThT solution and 100 μl aliquots from self-assembled or native antibodies suspensions were mixed together and measured immediately. Recombinant human insulin (Biological Industries, Beit Haemek, Israel) was used in parallel as a control peptide (data not shown). Self-assembled and native samples were tested every 30 or 60 min for the first 8 h and then daily up to 7 days at various antibodies concentrations from 1 to 5 mg/ml (data not shown). For further studies, working concentration was set for 1 mg/ml.
- *Circular dichroism (CD) spectroscopy*: IgG or IgY was prepared at concentration of 1 mg/ml in phosphate buffered saline (PBS) and kept under two pH conditions: physiological pH (control, native protein) and acidified (pH 2, self-assembled protein). Following 48 h incubation at 37 °C with gentle shaking, samples were diluted 50-fold in deionized water and analyzed by CD spectroscopy. Human recombinant insulin (at concentration of 1 mg/ml), native and self-assembled, was examined, as well as control. CD spectra were obtained using an Applied Photophysics Circular Dichroism Spectrometer equipped with a temperature-controlled sample holder and a 10-mm path length cuvette. For wavelength scan experiments, each spectrum represents the average of three scans from three independent samples.
- *Gel electrophoresis*: Samples of 1 mg/ml native or self-assembled IgG or IgY, as well as native or fibrillar insulin samples, were analyzed by SDS–PAGE in non-reducing conditions. The samples were loaded on a 12–16% polyacrylamide gel prepared in 1.5 M Tris–HCl, pH 8.8. The gradient gel was used to discern the whole IgG and IgY molecules (150 and 170 kD, respectively) from its possible cleaved subunits (25 and 50 kD, respectively) on the same gel. The samples were mixed with loading buffer [0.5 M Tris–HCl pH 6.8, 33% (v/v) glycerol, 3% (w/v) SDS, 0.5% (w/v) bromophenol blue] at room temperature and boiled for 3 min prior to loading. The gel was stained with Coomassie blue.
- *Atomic force microscopy (AFM)*: AFM was performed on IgG molecules absorbed onto mica surfaces. Twenty milliliters of stock IgG solution (1 mg/ml) diluted 1000- and 8000-fold in 10 mM Tris–acetate, pH 8.5, was incubated on a freshly cleaved muscovite mica plate for 5 min, washed with distilled water and dried with nitrogen gas. The native samples were diluted 8000-fold and not 1000-fold as in the case of the self-assembled preparations (see discussion for details). AFM images were obtained with a Solver PRO (NT-MDT, Moscow, Russia) AFM in semi-contact (tapping) mode using 130-mm long Si-gold-coated cantilevers (NT-MDT) with resonance frequency of 119–180 kHz. The images were visualized and analyzed using Nanotec Electronica S.L. (Madrid, Spain) WSxM imaging software (Horcas et al., 2007).

### Dissociation of self-assembled structures

Self-assembled or native IgG or IgY (both at concentration of 1 mg/ml) was diluted 100-fold (IgG) or serially up to 10 000-fold (IgY) in PBS at 4 °C with gentle shaking and tested for activity at various times after dilution, from time zero to 3 days.

### Ultracentrifugation

Soluble and non-soluble fractions were separated as follows: self-assembled or native IgG or IgY suspensions (1 ml volume, at concentration of 1 mg/ml) were ultracentrifuged (Sorvall Discovery M120 SE, Thermo Fisher Scientific, Waltham, MA; 300 000*g*, 120 min at 4 °C). Following centrifugation, the solutions were separated into the upper 900 μl supernatant and the lower 100 μl pellet. Fractions were frozen until further analysis.

### Cell culture

SKBR3, a human breast cancer cell line expressing HER2, was a kind gift from Prof. Canaani from the Biochemistry and Molecular Biology Department at Tel Aviv University, Israel. Cells were grown in McCoy’s 5A medium supplemented with 10% (v/v) fetal calf serum (FCS) and 1% (w/v) penicillin–streptomycin solution (Biological Industries).

### Fluorescence-activated cell sorting analysis

Fluorescence-activated cell sorting (FACS) procedures were carried out by FACScalibur (Becton Dickinson, Franklin Lakes, NJ). The FACS results were analyzed with WinMDI version 2.9 (Scripps Research Institute, La-Jolla, CA). Trypsinated SKBR3 cells were centrifuged, suspended and washed three times with FACS buffer: PBS with 0.1% (w/v) bovine serum albumin and 0.01% (w/v) sodium azide, and then incubated with 100 μL of dissociated self-assembled or native IgG solution (at *t* = 0 and up to 3 days dissociation) for 1 h, 4 °C with gentle shaking (300 000 cells per sample). After the first incubation, samples were washed three times with FACS buffer and incubated with goat anti-human Fc-FITC (Jackson Immunoresearch, Baltimore, PA) under the same conditions. Samples were analyzed by FACS after a further three washes with FACS buffer and buffer replacement with PBS.

### Competition assay

SKBR3 cells were trypsinized, washed (as in this section) and divided into tubes (300 000 cells/tube). Cells were incubated for 1 h at 4 °C with gentle shaking in the following solutions: (a) 100 μl PBS plus 100 μl NHS-FITC (Pierce Biotechnology, Waltham, MA) conjugated to IgG (1 mg/ml) as follows: 1 mg of NHS-fluorescein was reconstituted with 100 μl DMSO. NHS-fluorescein was mixed with antibody solution at 15:1 molar excess, and incubated for 2 h at room temperature. Excess NHS-fluorescein was removed by dialysis and the fluorescein-labeled protein was stored at 4 °C with sodium azide (0.1%) until use; (b) 100 μl Herceptin-FITC plus 100 μl dissociated self-assembled or native IgG (both at concentration of 10 μg/ml). Fluorescence intensities of the FITC-conjugated Herceptin in PBS were set as controls, while the decrease in fluorescence with the addition of native or dissociated self-assembled antibodies was attributed to the competitive binding of the non-fluorescent antibodies.

### Binding assay for IgY

The presence of antibodies against NDV antigen was determined by ELISA. Plates (Nunc, Waltham, MA) were incubated overnight at 4 °C with antigen diluted in carbonate-coating buffer (pH 9.6). Plates were washed following each step using washing buffer [0.05% (v/v) Tween–20 in PBS] then dried on a paper towel. Plates were blocked for 1 h at 37 °C. with 150 μl/well of blocking buffer [5% (w/v) skim milk dissolved in washing buffer]. Series of self-aggregated or native IgY dilutions were added and incubated for 1 h at 37 °C. Plates were then incubated (1 h in 37 °C) with horse radish peroxidase (HRP) conjugated to Rabbit anti-Chicken IgY secondary antibodies (Sigma-Aldrich, Rehovot, Israel), which were diluted 1:5000 in blocking buffer. Substrate solution of *o*-phenylenediamine dihydrochloride (Sigma–Aldrich) was added and optical density (OD) was determined at 450 nm by ELISA reader (Thermo Labsystems Multiscan RC, Waltham, MA). The titer was determined as the dilution at which the tested optical density reached that of the negative control.

### Statistical analysis

Data are expressed as mean ± standard deviation (SD). Statistical analysis of the data was performed using the two-tailed unequal variance Student *t*-test and *p* < 0.05 was considered significant.

## Results

### Ig’s aggregation: setting the conditions

ThT fluorescence at 482 nm is generally used to detect growing protein fibrils in solution. IgG and IgY at different concentrations (1–5 mg/ml) were examined with various ThT concentrations (0.5–500 μM) under the aggregation promoting conditions: gentle agitation (15 RPM) and harsh stirring, both at pH 2 and at 37 °C.

**Figure 1.**
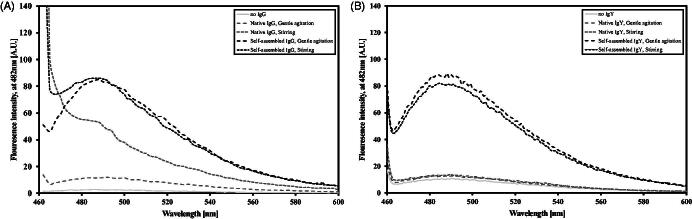
ThT spectra (100 μM) of native and self-assembled Igs (0.15 mg/ml) preparations. Preparation symbols are detailed in the inset box. (A) IgG and (B) IgY.

Figure 1 demonstrates the fluorescence spectra intensities of both IgG (Figure 1A) and IgY (Figure 1B) preparations, self-assembled and native, under different aggregation promoting conditions. The self-assembled preparations under gentle agitation produced a bell-shaped spectrum with a defined peak at 482 nm (Figure 1A). Similar IgG self-assembled preparation under stirring conditions lost its bell shape, though it still remained mostly similar to the gentle agitation curve. Both ThT alone and native IgG under gentle agitation conditions presented very low counts with no peak, native IgG preparation under stirring condition demonstrated a steep drop from its initial peak at 450 nm, similar to a turbid solution (as also evident to the naked eye). Thus, in all further preparations, the conditions for self-assembled IgGs were set to pH 2, 100 μM ThT and gentle agitation at 37 °C. Similar procedure with chicken IgY was performed yet the obtained spectra are fairly different. Both self-assembled preparations under gentle agitation or stirring demonstrated similar spectrum (bell-shaped spectrum with a defined peak at 482 nm) and both native formulations demonstrated similar spectrum (very low counts with a mild peak) (Figure 1B). As a control protein, insulin, which has been much investigated for its self-assembly properties in the past two decades, was used. Current findings recapitulate previous ones (Whittingham et al., 2002) (data not shown).

### Ig aggregation: characteristics

To gain more insights into the structure of the self-assembled immunoglobulins, complementing techniques were used including CD spectroscopy, gel electrophoresis and atomic forced microscopy (AFM).

**Figure 2.**
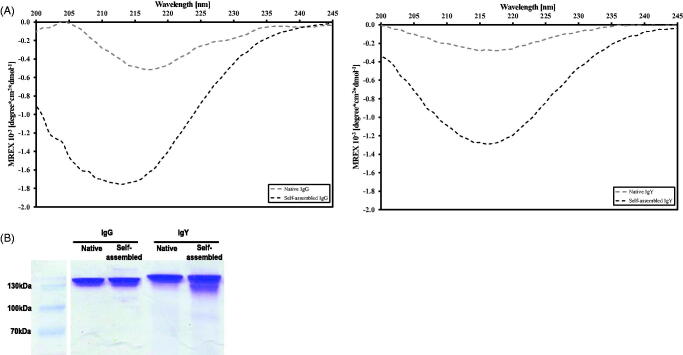
(A) CD spectra of native (gray) and self-assembled (black) IgG (left) and IgY (right) preparations (20 μg/ml). (B) Images of non-reducing gel electrophoresis for native and self-assembled IgG (left panel) and IgY (right panel).

A comparison of the native and self-assembled IgG preparations demonstrated different curve patterns: the latter showed a deepening and shift of the peak, with a significant negative peak in the 215–218 nm region indicative of an increase in beta-sheet folds (Figure 2A, left panel). IgY was examined under similar conditions and a deepening and shift of the peak was observed at the same region albeit the peaks are somewhat broader (Figure 2A, right panel).

To determine whether self-assembled antibodies remain intact during the aggregation process, self-assembled and native IgGs or IgYs (1 mg/ml concentration, 72 h preparation, 37 °C) were subjected to non-reducing gel electrophoresis. Bands of both self-assembled and native antibodies are located at their expected sizes of the complete molecules, 150 kD for IgG and 170 kD for IgY, with no apparent differences between the two treatments (Figure 2B).

**Figure 3.**
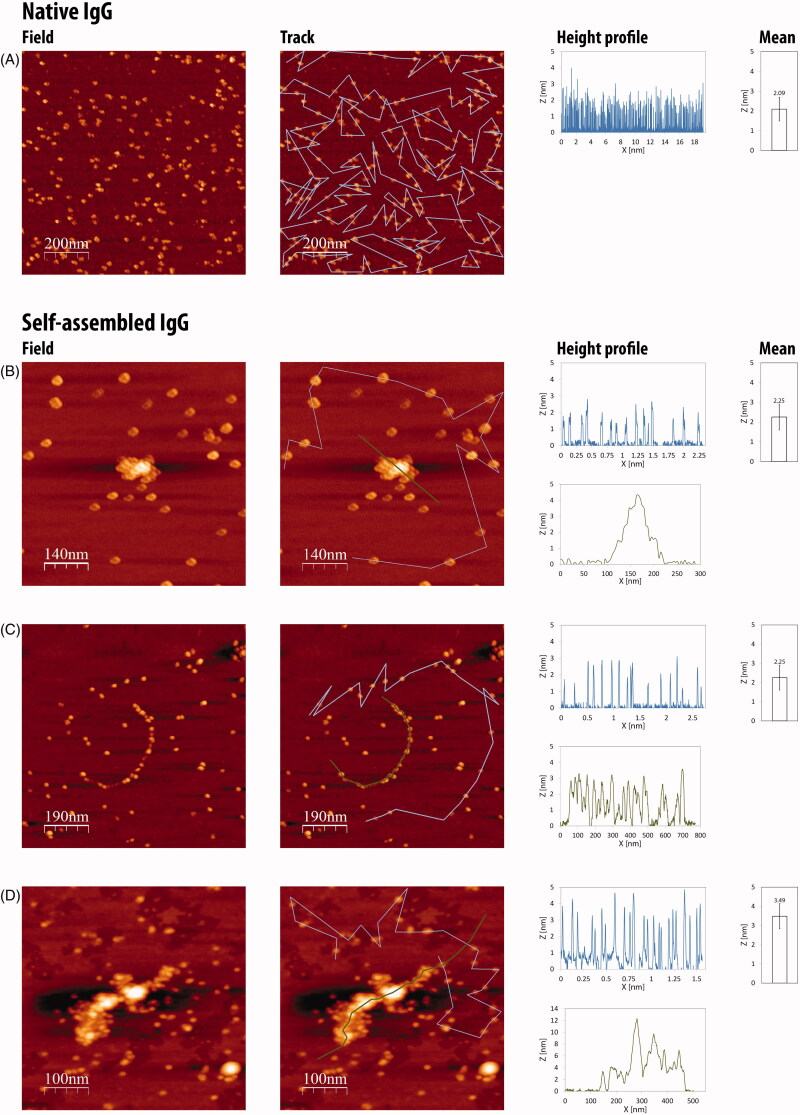
AFM images of native (A) versus self-assembled (B, 24 h; C, 48 h; D, 72 h preparation times) IgGs (1 mg/ml). Blue lines mark of discrete IgGs, green lines mark native IgG, all IgGs in the field were considered for height averaging. Native IgGs and self-assembled IgGs were diluted ×8000 and ×1000, respectively, prior to imaging. Bar graphs demonstrate mean height (±standard deviation) of measured IgGs in the field. Similarly, blue and green height charts are presented.

Native and self-assembled IgG (0, 24, 48 and 72 h preparations) were deposited on a mica surface and measured by AFM (see Methods). Native IgG molecules are characterized by height (mean 2.09 ± 0.6 nm) and shape, resembling a tri-nodular structure (Figure 3A, native IgG). Self-assembled IgG aggregates are composed of discrete antibodies yet the monomers are more rounded (Figure 3B–D, self-assembled IgG). These monomers self-assemble to form multimers which, in many cases, start from “strings” of antibodies in a row (Figure 3C, 48 h) and grow into larger multimeric structures composed of hundreds of monomers and having a length of up to 0.5 μM and a height of ∼12 nm (Figure 3D, 72 h). The monomers that make up the multimers are similar in size to the corresponding native monomers in the case of 24 h (1.98 ± 0.44 nm) and 48 h (2.25 ± 0.65 nm) preparations. The heights of the smallest IgG structures following 72 h preparation reach 3.49 ± 0.66 nm, which might indicate that these structures are IgGs dimers or even tetramers.

The proportion of multimers versus free antibodies in the treated preparations is assumed to be 80–90%. This assumption is based on the differences in dilution magnitudes between native and treated preparations. While attempting to view the native preparations in the 1000-fold dilution, we observed a white smear, indicative of a tight layer of antibodies attached to each other, preventing the differentiation of discrete molecules. Thus, a higher dilution (8000-fold) was required for the native preparations. The notion that in the treated preparations, a 1000-fold dilution is sufficient to differentiate between discrete antibodies and multimers demonstrate that most of the antibodies are within multimeric structures.

### Activity restoration and affinity of treated Ig’s

#### Binding activity of diluted multimers

**Figure 4.**
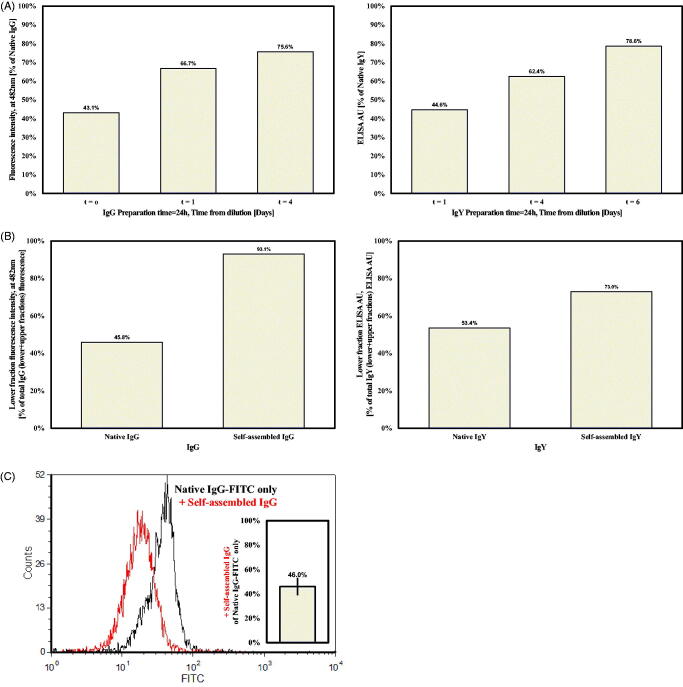
(A) Binding activity of self-assembled IgG (left panel) and IgY (right panel), following 24 h preparation (see text for dilution protocols). (B) Binding activity of lower fraction versus total binding activity) of 1 mg/ml IgG or IgY (left and right panels, respectively), after ultracentrifugation at 300 000*g*. (C) Competition assay for self-assembled versus native IgG binding to HER2 + expressing SKBR3 cells. FACS spectra of native IgG (black) versus native and self-assembled IgGs (red) binding capacity. The average drop in the native IgG binding activity is given in the bar graph.

To examine the possibility that aggregated IgG or IgY multimers can release active antibodies and restore biological activity, samples of self-assembled (24 and 72 h preparation time) and native Igs were diluted 1:100 in PBS and tested for their binding capacity over 3 days in a binding assay to HER2-positive SKBR3 human breast cancer cells or to Newcastle disease virus (NDV) antigen, respectively. In this work, *t* = 0 is actually 2 h after dilution due to incubation period and subsequent washes. It is evident that restoration of binding activity is time-dependent, for both IgG and IgY self-assembled preparation (as compared to native preparations). In the case of IgG, approximately 75–80% of the total IgG and IgY binding activities was restored 4 and 6 days into the process, respectively (Figure 4A). In the case of the longer preparation time (72 h), similar results were obtained yet the time until 80% restoration was 20–25% longer (data not shown).

#### Binding activity of thermodynamically separated self-assembled Igs versus Igs monomers

To further establish the existence of aggregates and their ability to dissociate and release active IgG, a thermodynamic approach was applied. Samples of 1 ml self-assembled/native (3 days preparation) Ig’s were centrifuged at 300 000*g* for 2 h at 4 °C. After centrifugation, the upper 900 μl fraction from each tube was removed and considered as “supernatant”, while the remaining lower l00 μl fraction was considered as “pellet”. Fractions were diluted according to our protocol for 72 h prior to a binding assay. While most (93%) of self-assembled IgG binding activity was attributed to the pellet, the native IgG’s binding activity was as expected split almost evenly between the pellet and supernatant (Figure 4B, left panel). Almost similar results were obtained for IgY, where 73% of self-assembled IgY binding activity was attributed to the pellet, and native IgY’s binding activity was similar in both fractions (Figure 4B, right panel).

#### Availability of released IgG’s from multimers

To test the binding availability of dissociated antibodies, a competition assay between native and self-assembled IgG and a FITC-conjugated native IgG was performed. Multimers were prepared for 3 days, followed by a 3-day dissociation period prior to the experiment. Multimeric preparations were mixed with equal amounts of FITC-conjugated native IgG and incubated with 300 000 SKBR3 cells. FITC-conjugated Herceptin fluorescence was decreased by half following the addition of multimeric IgG (Figure 4C, to mean level of 46%±6.5% of control FITC-conjugated Herceptin only). Similar findings were obtained also when native IgG was added (data not shown). This phenomenon was not dependent on IgG concentrations (at the range of 5–10 μg, data not shown), indicating comparable binding availabilities between the native IgG and the self-assembled released monomers.

#### Monomers release after long incubation periods

First, restoration of multimers activity was tested after longer incubation period in harsh condition. Multimers and native IgG were kept at 60 °C with shaking for 2 months. Two dilutions were performed at *t* = 0 and 3 days. Both multimeric and native IgGs lost their binding ability (data not shown).

**Figure 5.**
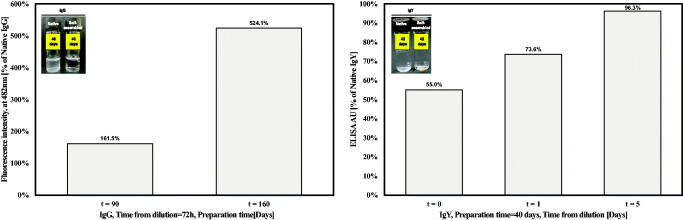
Binding activity after long incubation periods of 1 mg/ml self-assembled IgG (left panel) and IgY (right panel). Binding is compared to the native antibodies binding capacity under similar conditions. Images in the inset panel represent vails containing the native (left vail) and self-assembled (right vail) suspensions after 40 (IgG) or 42 (IgY) days.

Then, it was also tested under less detrimental condition. In the case of IgG, after 40 days incubation of both self-assembled and native formulations, it was apparent to naked eyes that only the native formulation became turbid (Figure 5A, left panel), yet it retained its full biological activity, compared to freshly dilute antibody, after 3 days dilution (data not shown). Prolonged preparation periods of 90 and 160 days (from *t* = 0) revealed that the self-assembled formulations had higher binding activity compared to the native ones, by 60% and 500%, respectively (Figure 5B, left panel). In the case of IgY, no apparent turbidity was observed in both self-assembled and native preparations after 42 days (Figure 5B, left panel). Binding activity of self-assembled IgY was half of the native form immediately after dilution, and gradually increased until full restoration after only 5 days from dilution (Figure 5B, right panel).

## Discussion

Antibodies assembly has been reported as the cause of several human ailments, mostly due to light-chain aggregation in different tissues (Ionescu-Zanetti et al., 1999). Biophysical studies have been performed to monitor the thermodynamics and kinetics of human monoclonal IgG aggregation, and a kinetic model for the process was proposed (Brummitt et al., 2011a,b). Protein fibrillation, or self-assembly, can be viewed as a biotechnological opportunity (Dekel et al., 2010). The use of aggregated protein structures as a protein depository has been reported to occur *in vivo* in rats’ brains (Maji et al., 2009). The ability of insulin to form self-assembled fibrillar structures and release active monomers, under the appropriate conditions, has been reported both *in vitro* and *in vivo* (Dekel et al., 2010). Due to their ordered protected structure and their innate ability to dissociate and restore biological activity, self-assembled protein aggregates are of high interest to protein formulators and biomaterial scientists.

In this study, Herceptin – a 150-kD human monoclonal IgG and the first line of treatment in HER2-positive breast cancer, and chicken IgY – an immensely important Ig in the poultry industry, were driven to a multimeric state at pH 2. The self-assembly process was monitored under different conditions, using the fluorescent dye ThT. Fluorescence intensity was concentration-dependent, on both IgG concentration (from 1 to 5 mg/ml) – similarly to other proteins that undergo ordered aggregation (Nielsen et al., 2001), and ThT concentration (at the range of 4 μM to 100 μM) (data not shown) – a characteristic of HMWPs (Ramshini et al., 2011), rather than amyloidogenic self-fibrillation (Kelly, 1996) (data not shown). The steep drop in ThT fluorescence at higher concentration (500 μM, data not shown) may be a result of non-specific interactions, as has been observed with other surfactants (Friedman & Caflisch, 2011).

The self-assembly process was conducted at 37 °C. It is well established that at higher temperatures, up to 70 °C, the self-assembly or fibrillation kinetics of many proteins accelerates (Nielsen et al., 2001). However, the end goal of this research was to enable restoration of biological activity and to that end, a physiological temperature was applied. Agitation is another enhancer of protein aggregation. While attempting to form self-assembled IgG structures under gentle agitation or stirring conditions, the shape of the ThT fluorescent curve changed from its regular “bell shape” to a more pointed shape. A turbid solution evolved in the case of the control native IgG under stirring, as also supported by the shape of its ThT curve (Figure 1A). In the case of the self-assembled IgG, the solution is probably composed of amorphous aggregates and ordered structures, as also supported by the curve shape which represents a combination of both ordered and unordered structures. Self-assembled IgY preparations, both under gentle agitation and stirring conditions, showed a fairly similar spectrum with a peak at 482 nm, same as for the self-assembled IgG in gentle agitation and in similar fluorescence intensities. Nevertheless, in contrast to self-assembled IgG under stirring conditions, the corresponding IgY solution was not turbid. This observation is correlated with the bell shape spectra. The reason for that might be related to the polyclonal nature of IgY and the process of its production from eggs in which IgY is enriched in the outcome yet other proteins might exist in the extracted solution as well (Aizenshtein et al., 2016). One of the factors that dictate protein ordered association kinetics is the purity of the batch. Small differences in protein sequence can have dramatic effects on ordered aggregates formation (Pashley et al., 2016).

To track conformational changes, the far UV spectra of self-assembly and native Igs were compared by CD analysis (Kardos et al., 2011). The ordered aggregation or assembly process in general is characterized by an alteration from the native fold toward different conformers with high beta-sheet content. In the case of insulin, for example the native form is composed of 95% alpha-helical structures, whereas in the aggregate form, a clear transition in the secondary structure was observed toward a single peak ranging from 215 to 220 nm indicative of beta-sheet secondary structures similar to the aggregate state observed with IgG/IgY (Hosseinzadeh et al., 2016). An evident increase in the amplitude of a negative peak (215 and 220 nm, Figure 2) was obtained for the self-assembled Igs (pH 2), compared to their native state (pH 7), which indicates a shift in the secondary structure toward a predominantly beta-sheet structure (Jiang et al., 2012). Altogether, the CD spectra and ThT fluorescence results suggest that the Igs aggregation process under the current condition involves conformational change of monomers toward beta-sheet structures.

Another important aspect is to determine if the proteins remain intact within the aggregated form. Thus, proteins are usually exposed to harsh conditions such as very low pH (the makeup process) and boiling. These are regular procedures when preparing proteins for gel electrophoresis. Self-assembled and native Igs were subjected to a non-reducing gel electrophoresis, in order to evaluate the overall completeness of the Igs molecules. Both formulations remained at the expected size for intact IgG/IgY (150 /170 kD, respectively) with similar bands intensities (Figure 2B). Beta-lactoglobulin has been reported to form fibrillar structures when heated to 80 °C but when subjected to gel electrophoresis, the self-assembled structures dissociate into different peptides as the main beta-lactoglobulin band fades and smaller bands appear (Oboroceanu et al., 2010). The authors concluded that during the formation of fibrillar structures, beta-lactoglobulin dissociates into smaller peptides that form fibrils. Here, no significant dissociation to other peptide species was apparent; this might have been due to the preparation procedure, in which a physiological temperature was applied. The results suggest that the structures are formed from whole IgG/IgY molecules and not from dissociated peptides.

AFM imaging provided direct evidence of the formation of multimeric structures made up of different numbers of discrete IgG monomers. Native IgG molecules have a height of 2.09 nm in average, which agrees well with earlier published data (Thomson, 2005a,b). The shape of the IgG units in the self-assembled preparations is different from that of native IgG molecules (Figure 3A compared to Figure 3B–D). This alteration in shape might be due to a transition in conformation of the IgG molecule, a shift that has been reported for other proteins under acidic pH conditions and was also observed in the CD spectra inhere (Pedersen & Otzen, 2008). The discrete IgGs join together and form dimers, tetramers and high-order structures. The buildup of the multimers is gradual. Relatively small structures (4.5 nm in height) can be observed already after 24 h with where bigger ones (12 nm in height and 0.5 μM in length) were detected after 72 h. After 48 h structures that resemble multimeric strings of spherical monomers were observed, and these strings might be the backbone for the larger structures that are seen after 72 h (Figure 3C and D). Another important observation is related to the height (3.49 nm) of the smallest structures in the 72 h preparation in comparison with the shorter times (averaging 2 nm). This might indicate that at the longer preparation time, there are almost no monomers and all antibodies are at least in structures of dimers/trimers. It is well established that aromatic amino acids are crucial for the self-assembly of proteins, peptides and discrete amino acids (Soreq & Gazit, 2008). Both heavy and light chains of Herceptin contain aromatic residues (Babor et al., 2011). In light of the results here that dilution of self-assembled IgG solutions results in the release of monomers from multimers, one can speculate that some of the monomers, dimers or other smaller entities, which were apparent in the images, were formed during the 1000-fold dilution, prior to imaging.

Two main approaches are applicable in the case of protein aggregates dissociation: (a) active dissociation by applying harsh conditions such as boiling temperature or high concentrations of urea or guanidine hydrochloride (the usual practices to dissociate self-assembled protein structures). Examples have been reported with beta-microglobulin and lactoglobulin fibrillar structures that can dissociate at boiling temperatures, in acid or in different ethanol mixtures (Jordens et al., 2011; Kardos et al., 2011); (b) converting the thermodynamic state from that favoring aggregation (high protein concentration, low pH) to non-favoring aggregation conditions (low protein concentration, physiological pH) by diluting the protein solution in physiological buffer. The second approach was applied so as not to damage the Igs and regain biological activity from the multimers. This was done by diluting the solution of self-assembled structures.

Dissociation of active IgG units was confirmed with a binding assay to SKBR3 cells by FACS. Relative binding capacity (compared to native IgG), increased gradually from 40% to 67% and 75% at *t* = 0, 1 and 4 days of dilution, respectively. Similarly, binding of self-assembled IgY to NDV antigen was assessed by ELISA, and increased gradually to reach 80% after 6 days. These results indicate that the multimeric structures released active antibodies in a dilution/time-dependent manner. Vast research on Ig’s aggregation kinetics was previously performed, yet at different aggregation makeup conditions. It was reported that under conditions of 70 °C the aggregation continues beyond dimers irreversibly and dilution is not capable of dissociating the aggregates at several pH values. The authors suggested that at lower temperatures the aggregation might proceed with a native aggregation mechanism (Nicoud et al., 2016). This suggestion is in agreement with our results.

To further establish that the active Ig fraction originate from aggregates and not from mixtures of native Igs and aggregates, a thermodynamic separation with ultracentrifugation was performed. The supernatant and pellet from each sample were subjected to its suitable binding assay. While there was no difference between the binding capacity of the supernatant and pellet fractions for the native forms of both IgG and IgY, most of the binding capacity of the self-assembled preparations was within the pellet fraction of IgG (93%) and IgY (73%) (Figure 4C, left panel). This clearly demonstrates that most active antibodies originated from the self-assembled entities that sediment following centrifugation. This notion is compatible with our above-described observation during sample preparation for AFM. The differences between IgG and IgY may be attributed to the rate of aggregation, and the nature and purity of the Abs (monoclonal versus polyclonal, respectively), as explained before.

A competition assay between native FITC-tagged IgGs and self-assembled IgGs revealed comparable binding capacity (Figure 4D). This indicates that the antibodies released from the self-assembled multimers are active and can compete with native antibodies for the shared epitope. Combining these results with those from the electrophoresis test, one can speculate that no alteration in IgG structure occurred during the assembly to multimers and release from them, such that IgG function was not affected.

In general, ordered protein aggregates are stable under harsh conditions and thus might enable better preservation of the proteins when compared to regular native formulations (Jimenez et al., 2002). A crucial aspect of every protein formulation is its stability under aggregate-favoring conditions, mainly heat and shaking (Lee et al., 2005). Here, restoration of activity after long incubation period was tested under both harsh and less detrimental condition. Both IgG preparations lost their binding activity under harsh conditions (data not shown). Yet, under permissible conditions self-assembled IgG retained its activity, to a much higher extent compared to native IgG and for a longer period (Figure 5B). In addition, the activity of self-assembled IgY was comparable to native one, while being restored in a dilution time-dependent manner (Figure 5B), resembling its activity after a short incubation time (Figure 4A). This observation is in agreement with reports on IgY stability under extreme pH conditions or even to proteinases (Rahman et al., 2013).

## Conclusions

Taken together, our results indicate that both IgG and IgY can form self-assembled multimers from discrete native antibodies under low pH and gentle agitation. Active antibodies are released in a sustained manner from the multimers, while retaining their activity and biological availability throughout the process. The ability to release active antibodies is dependent on the multimeric status, might influence the long-term stability and enable a greater shelf life. The multimers are unique in that they serve as a storage depository, release active antibodies and protect the antibodies composing them, thereby serving self-delivery entities. In the future, these three important functions can provide proteins formulations with the characteristics needed for novel administration protocols.
